# Pretreatment chest x-ray severity and its relation to bacterial burden in smear positive pulmonary tuberculosis

**DOI:** 10.1186/s12916-018-1053-3

**Published:** 2018-05-21

**Authors:** S. E. Murthy, F. Chatterjee, A. Crook, R. Dawson, C. Mendel, M. E. Murphy, S. R. Murray, A. J. Nunn, P. P. J. Phillips, Kasha P. Singh, T. D. McHugh, S. H. Gillespie, On behalf of the REMoxTB Consortium, Andreas Diacon, Madeleine Hanekom, Amour Venter, Kimberley Narunsky, B. Mtafya, N. Elias Ntinginya, Andrea Rachow, Evans Amukoye, B. Miheso, M. Njoroje, Noel Sam, D. Damas, Alphonce Liyoyo, A. Ahmad Mahayiddin, C. Chuchottaworn, J. Boonyasopun, B. Saipan, Shabir Lakhi, D. Chanda, J. Mcyeze, Alexander Pym, N. Ngcobo, Cheryl Louw, H. Veldsman, Gerardo Amaya-Tapia, T. Vejar Aguirre, D. K. Chauhan, R. K. Garg, N. K. Jain, A. Aggarwal, M. Mishra, S. Teotia, S. Charalambous, N. Hattidge, L. Pretorious, N. Padayachi, L. Mohapi, M. Gao, X. Li, L. Zhang, Q. Zhang, S. Aggarwal, Ketty Belizaire, Majda Benhayoun, D. Everitt, Ann Ginsberg, Martino Laurenzi, Bridget Rawls, Christopher Radali, Mel Spigelman, Almarie Uys, Christo van Niekerk, Anna L. C. Bateson, Matthew Betteridge, S. Birkby, Emily Bongard, Michael Brown, Holly Ciesielczuk, C. Cook, E. Cunningham, James Huggett, Robert Hunt, Clare Ling, Marc Lipman, Paul Mee, Felicity M. R. Perrin, Robert Shorten, K. Smith, Victoria Yorke-Edwards, Alimuddin Zumla

**Affiliations:** 1 UCL Centre for Clinical Microbiology, Department of Infection, University College London, Royal Free Campus, Rowland Hill Street, London, NW3 2PF UK; 2 Department of Radiology, Barts Health NHS Trust, The Royal London Hospital, Whitechapel Road, London, E1 1BB UK; 3 Medical Research Council UK Clinical Trials Unit at University College London, Aviation House, 125 Kingsway, London, WC2B 6NH UK; 4 University of Cape Town Lung Institute, George Street, Mowbray, Cape Town, South Africa; 5 Global Alliance for Tuberculosis Drug Development, New York, NY 10005 USA; 6 Medical and Biological Sciences, School of Medicine, University of St Andrews, North Haugh, St Andrews, KY16 9TF UK

**Keywords:** Pulmonary tuberculosis, chest x-ray, cavitation, pretreatment

## Abstract

**Background:**

Chest radiographs are used for diagnosis and severity assessment in tuberculosis (TB). The extent of disease as determined by smear grade and cavitation as a binary measure can predict 2-month smear results, but little has been done to determine whether radiological severity reflects the bacterial burden at diagnosis.

**Methods:**

Pre-treatment chest x-rays from 1837 participants with smear-positive pulmonary TB enrolled into the REMoxTB trial (Gillespie et al., N Engl J Med 371:1577–87, 2014) were retrospectively reviewed. Two clinicians blinded to clinical details using the Ralph scoring system performed separate readings. An independent reader reviewed discrepant results for quality assessment and cavity presence. Cavitation presence was plotted against time to positivity (TTP) of sputum liquid cultures (MGIT 960). The Wilcoxon rank sum test was performed to calculate the difference in average TTP for these groups. The average lung field affected was compared to log _10_ TTP by linear regression. Baseline markers of disease severity and patient characteristics were added in univariable regression analysis against radiological severity and a multivariable regression model was created to explore their relationship.

**Results:**

For 1354 participants, the median TTP was 117 h (4.88 days), being 26 h longer (95% CI 16–30, *p* < 0.001) in patients without cavitation compared to those with cavitation. The median percentage of lung-field affected was 18.1% (IQR 11.3–28.8%). For every 10-fold increase in TTP, the area of lung field affected decreased by 11.4%. Multivariable models showed that serum albumin decreased significantly as the percentage of lung field area increased in both those with and without cavitation. In addition, BMI and logged TTP had a small but significant effect in those with cavitation and the number of severe TB symptoms in the non-cavitation group also had a small effect, whilst other factors found to be significant on univariable analysis lost this effect in the model.

**Conclusions:**

The radiological severity of disease on chest x-ray prior to treatment in smear positive pulmonary TB patients is weakly associated with the bacterial burden. When compared against other variables at diagnosis, this effect is lost in those without cavitation. Radiological severity does reflect the overall disease severity in smear positive pulmonary TB, but we suggest that clinicians should be cautious in over-interpreting the significance of radiological disease extent at diagnosis.

**Electronic supplementary material:**

The online version of this article (10.1186/s12916-018-1053-3) contains supplementary material, which is available to authorized users.

## Background

Since their introduction in routine clinical practice in the 1920s, chest radiographs have been used as a primary tool to diagnose and manage pulmonary tuberculosis (PTB) [1–3]. To date, despite their limitations and the availability of computed tomography, they remain the most commonly used tool in PTB diagnosis and management worldwide [4–6]. The chest x-ray (CXR) has been used not only as a diagnostic tool, but also to estimate disease severity in multiple TB studies and clinical trials [7–9].

There are several methods of grading the radiological severity of disease by estimating the extent of lung field that is ‘abnormal’, including the WHO grading system [10] or the US National Tuberculosis and Respiratory Disease Association classification [11]. In 2010, Ralph et al. [12] created a simple validated scoring system (using a score out of 140) from findings that the proportion of lung fields affected by disease at diagnosis of PTB was associated with a greater acid fast bacilli (AFB) smear grade and that the presence of cavitation (but not number or size of cavitation), along with the percentage of lung field affected on CXR, predicted 2-month smear positivity on treatment. This has gained some currency in studies describing radiological severity [13–15] and in a subsequent study that validated this approach [16].

The relationship between radiological appearance and disease severity has been assessed by comparison with measures of bacterial load such as smear microscopy and culture [17–21]. At diagnosis, the presence of cavitation visible on CXR has been associated with a higher sputum AFB smear grade [12, 17, 22]. The time taken for specimens in automated liquid culture to signal positive is inversely related to bacterial load [13, 23, 24]. Using this concept, a study of 95 images showed that the presence of cavitation on CXR was associated with a shorter time to positivity (TTP) [21]. More recently, some studies have shown that patients with cavitation have a higher bacterial load as judged by TTP in liquid culture [23, 24]. Another study of 244 patients with radiographic assessment of cavitation found that the colony forming units per milliliter were significantly higher in those with cavitation; this was also true using TTP as a marker for bacterial load [22].

In his seminal review of post mortem examinations of patients with TB, Canetti [25] described a difference in the number of bacteria in lung tissue of samples with cavities compared to those with caseous necrotic tissue only and areas of alveolitis. He found that tubercle bacilli were abundant in the inner layer of a cavity, abundant but less so in a solid area of caseous tissue, and rare within areas of inflammatory tissue. As Canetti described bacilli-rich areas as well as areas of inflammatory change, one would think that the host’s inflammatory response in addition to the bacterial load should affect the findings on the CXR prior to treatment. We look at a number of patient factors that may affect this host response and which have been associated with radiological findings in other studies, such as on HIV status [26], diabetes mellitus status [27, 28], age [29], ethnicity [30, 31], and gender [32], to investigate what host factors affect radiological severity. Hypoalbuminemia at diagnosis of PTB and low body mass index (BMI) are surrogates of disease severity known to lower survival rates [33, 34]. TB symptoms at diagnosis have been associated with worse burden of disease [35].

With so much weight put on the extent of radiological findings, little is known about what this reflects. We use the REMoxTB database [36] of patients from Africa and Asia with PTB to determine whether radiological extent of disease judged by the CXR severity score correlates with *M. tuberculosis* bacterial load as measured by Mycobacteria-Growth-Indicator-Tube (MGIT) TTP.

## Methods

### Study sites and patients

Data were collected from the REMoxTB clinical trial, which compared the use of two 4-month moxifloxacin-containing regimens to the standard 6 month first line treatment for PTB [36]. Between 2007 and 2012, 1931 patients were enrolled from 51 sites across 8 countries in Africa and Asia and the protocol mandated pre-treatment postero-anterior CXRs, sputum sampling for AFB smear and culture, and routine blood tests (including liver function tests, albumin levels and HIV testing). During the trial, study patients were excluded if they had severe medical comorbidities or were already taking antiretroviral treatment for HIV prior to study enrollment. In this study, all patients were adults aged 18 years or more, who had smear- and culture-positive PTB by molecular speciation.

### CXR scoring

The CXR images were taken at the clinical sites by a radiographer and either uploaded as a digital image (DICOM file) or presented to the clinical site staff as a plain film. Plain films were digitalized with digital photography using a standard protocol to ensure images were of an adequate quality. An early assessment of ‘readability’ was performed and, where films were judged poor, sites were asked to re-take the images. All images were converted into DICOM files for evaluation.

The digital images were read independently by two clinicians (SHG and SEM) using the Osirix medical imaging software on Apple iMAC computers with at least 1920 × 1080 pixel screens, and readers were encouraged to take regular breaks during the reading process. Images were sent to readers by study site and were read in the same order.

Both readers followed standardized criteria to establish whether an image was of sufficient quality for analysis (Table 1). If deemed satisfactory, the image was assessed for the presence of cavitation and a measure of percentage of abnormal lung field. In the case of discrepant results on readability or presence of cavitation, a third reader blinded to the primary assessment reviewed the film (FC). Only those images that the first two readers agreed on for readability or those that the third reader deemed readable were used in the final analysis. The final result for cavity presence was based on agreement between the primary readers or, if discrepant, the majority result including the third reading. The percentage of lung field affected was calculated using the method described by Ralph et al. [12], where the reader divides the lung fields into quadrants and by observation scores each quadrant by its percentage of abnormal opacification. The scores are then added together and divided by four to produce a total percentage of lung field affected by disease.

**Table 1:** Inclusion and exclusion criteria for deeming an image of sufficient quality for reading

Inclusion	Exclusion
Postero-anterior film	Artefacts obscuring the view of the lung fields
A full view of lung fields – the whole thorax with the first rib, lateral ribs and costophrenic angles in view	Images acquired more than 4 weeks prior to pretreatment visit or more than 3 weeks after this
Adequate penetration of film to allow ribs and lung parenchyma to be distinguished	

### Microbiological and clinical data

Sputum samples and demographic data were collected as part of the clinical trial protocol at screening and baseline visits, prior to starting treatment. Sputum samples were either early morning samples or spot samples, none of which were induced. The samples were processed by standard methodology and graded as described in the trial report. Samples that were re-treated due to contamination were not included in the analysis as this process altered the calculated TTP and, thus, could not guarantee an accurate quantification result. As part of the pre-treatment assessment, participants were tested for HIV and were asked about a history of diabetes mellitus. In addition, a series of questions about symptoms were asked and symptoms graded by severity using the modified Division of AIDS system [37] (Table 2).

**Table 2:** Division of AIDS (DAIDS) grading of adverse event (AE) severity (modified version). This describes the grading system referred to in this study to describe the severity of TB symptoms such as cough, night sweats, weight loss, and hemoptysis

Parameter	Grade 1	Grade 2	Grade 3	Grade 4
Clinical AE NOT identified elsewhere in this DAIDS AE grading table	Symptoms causing no or minimal interference with usual social and functional activities	Symptoms causing greater than minimal interference with usual social and functional activities	Symptoms causing inability to perform usual social and functional activities	Symptoms causing inability to perform basic self-care functions OR Medical or operative intervention indicated to prevent permanent impairment, persistent disability, or death
e.g., Unintentional weight loss	NA	5–9% loss in body weight from baseline	10–19% loss in body weight from baseline	≥ 20% loss in body weight from baseline OR aggressive intervention indicated (e.g., tube feeding or total parenteral nutrition)

### Statistical analysis

The inter-reader variability was presented on a Bland–Altman plot using the final severity scores from readers 1 and 2. The average of the two readers’ calculation of the percentage of lung field affected was used with the final results of cavity assessment. Images where readers disagreed by 1.96 standard deviations or more were not included in the analysis to ensure accuracy in the average percentage value. The presence or absence of cavitation was plotted against TTP and a Wilcoxon rank sum test to calculate the difference in average TTP for each of the two groups was performed. The average percentage area of lung field affected was compared to log_10_TTP using linear regression and plotted on a scatterplot.

Baseline clinical and biochemical findings (age, sex, ethnicity, BMI, serum albumin, number of grade 3 or 4 TB symptoms, HIV status and type II diabetes status) and radiological severity score were included in a univariable regression analysis. Those found to be significant (*p* < 0.05) were used to create a multivariable regression model to determine the relationship of these characteristics with the radiological severity score. For this process the participants were put into two groups; those with cavitation and those without. Wilcoxon rank sum tests and χ^2^ tests were used to compare both groups. All statistical analysis was performed using R statistical software [38].

### Ethical approval

This study was performed within the scope of the approvals provided for the REMoxTB clinical trial [36].

## Results

**Fig. 1:**
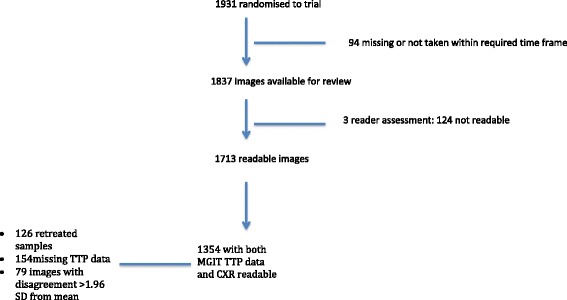
Flow diagram showing breakdown of final cohort for analysis

Out of 1931 patients randomized for the trial, 1837 had CXRs taken within the required protocol time frame. Following the three-reader quality assessment, 1713 images were deemed readable. Taking into account available data required for analysis, including non-retreated culture results with TTP data, the total number of cases was 1354 from 47 study sites (Fig. 1). The baseline characteristics and findings for the 1354 cases with available matching data are shown in Table 3 and breakdown of participants by site in Table 4. A comparison of the characteristics between the included and excluded cohort are also shown to ensure sampling bias was not an issue (Table 5).

**Table 3:** Baseline characteristics of final 1354 subjects

Baseline characteristic	N (%)	Median	IQR (1st, 3rd quartile)
Age (years)		31.6	24.1, 41.9
Sex			
Male	959 (70.8%)		
Female	395 (29.2%)		
Ethnicity			
African	924 (68.2%)		
Asian	430 (31.8%)		
Diabetes			
Yes	69 (5%)		
No	1285 (95%)		
HIV			
Yes	104 (7.7%)		
No	1250 (92.3%)		
Albumin (g/L)		34	30, 38
Body mass index (kg/m^2^)		18.5	16.9, 20.3
Grade 3+ tuberculosis symptoms			
0	1271 (93.9%)		
1	60 (4.4%)		
2	16 (1.2%)		
3	7 (0.5%)		
4	1 (0.07%)		
Time to positivity (hours)		117	89, 153
Cavity			
Yes	1049 (77.5%)		
No	305 (22.5%)		
Average area affected (%)		18.13	11.25, 27.5

**Table 4:** The 47 sites across 8 countries where the participants (1354) were recruited

COUNTRY	SITE	N (total 1354)
South Africa	Stellenbosch	325
	Cape Town	221
	Durban	36
	Johannesburg	73
	Durban 2	7
	Soweto	1
	Ukzn	4
	Brits	48
	Tembisa	4
Tanzania	Moshi	57
	Mbeya	72
Zambia	Lusaka	40
Kenya	Nairobi	36
Thailand (2 sites)	Bangkok	104
Malaysia	Kuala Lumpur	56
China	Tianjin	18
India (29 sites)	Dehli, Agra, Jaipur	252

**Table 5:** A comparison of the included and excluded cohorts. Using χ^2^ tests and Wilcoxon rank sum test *p* values are provided

	Included (*n* = 1354)			Excluded (*n* = 359)			
Baseline characteristic	N (%)	Median	IQR (1st, 3rd quartile)	N (%)	Median	IQR (1st, 3rd quartile)	*p* value
Age (years)		31.6	24.1, 41.9		29.8	24.2, 40.8	0.39
Body mass index (kg/m^2^)		18.5	16.9, 20.3		18.2	16.3, 20.3	0.06
Sex							0.32
Male	959(70.8%)			244 (68%)			
Female	395 (29.2%)			115 (32%)			
Ethnicity							1.0
African	924 (68.2%)			245 (68.2%)			
Asian	430 (31.8%)			114 (31.8%)			
Diabetes							0.30
Yes	69 (5%)			13 (3.6%)			
No	1285 (95%)			346 (96.4%)			
HIV							0.10
Yes	104 (7.7%)			18 (5%)			
No	1250 (92.3%)			341 (95%)			
Albumin (g/L)		34	30, 38		35	30, 39.3	0.19
Time to positivity (hours)		117	89, 153	(*n* = 79 with values)	105	85.5, 140.5	0.10
Cavity							1.0
Yes	1049 (77.5%)			278 (77.4%)			
No	305 (22.5%)			81 (22.6%)			
Average area affected		18.13	11.3, 27.5		21.88	11.9, 32.5	0.001

### Reader agreement

**Fig. 2:**
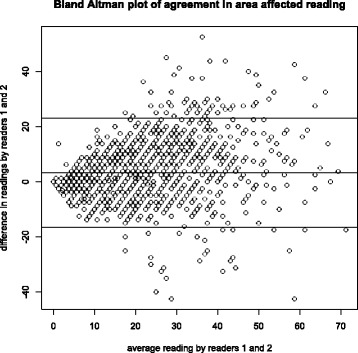
Bland–Altman plot demonstrating the level of agreement between readers 1 and 2 in scoring the 1713 images for radiological severity (x axis: the mean average numerical score between readers 1 and 2, y axis: the difference in scores for each image between readers 1 and 2). Horizontal lines show the mean ± 1.96 standard deviations; 3.34 (23.11−16.44) (SD = 10.10)

There was agreement for 1394 (76%) of the 1837 images available for either their readable quality or the presence or absence of cavitation. Agreement between the two readers on cavitation presence was 0.495 by Cohen’s Kappa score (95% CI 0.45–0.54, *p* < 0.001), where a value of < 0.4 is poor, 0.4–0.75 is fair to good, and > 0.75 to 1 is excellent [39]. The level of agreement when assessing the percentage of the area of lung field affected was illustrated using a Bland–Altman plot (Fig. 2).

### Cavity presence and bacterial load

**Fig. 3:**
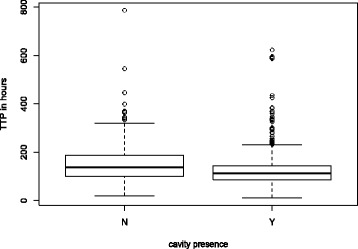
Boxplot of TTP distribution comparing subjects without and those with cavitation present on CXR. Thick black horizontal lines represent the median values with the interquartile range being the horizontal edges of the boxes. The overall range lies out with these and extreme outliers above the plots

The number of images confirmed to have cavitation visible was 1049 (77.5%) of 1354. The median TTP for MGIT samples from all 1354 patients was 117 h (4.88 days) with an interquartile range of 89 h (3.7 days) to 153 h (6.4 days). Figure 3 shows a boxplot of distribution of TTP between those without and with cavitation on CXR at baseline. This demonstrates that the median TTP is 26 h greater in those patients without compared to those with cavitation (95% CI 16–30, *p* < 0.001, Wilcoxon rank sum test).

### Extent of radiological disease and bacterial load

**Fig. 4:**
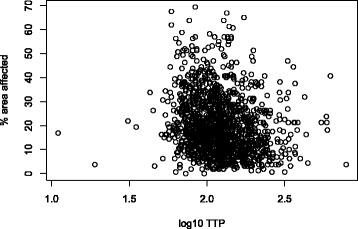
Scatterplot showing the log_10_TTP (hours) from baseline sputum cultures against the percentage of lung field affected on the CXRs

The median percentage of lung fields affected on the chest radiographs was 18.1% (interquartile range 11.3–27.5%). Figure 4 shows a scatterplot of the percentage of lung field affected against the sputum culture log_10_TTP values for the 1354 patients. Using linear regression for every 10-fold increase in TTP, the area affected decreases by 11.4% (*p* < 0.001, 95% CI 14.9–7.9%).

### Multivariable regression model: pre-treatment factors and baseline radiological severity

The percentage of lung fields affected was compared to other parameters in two groups; those with cavity presence and those without cavitation. Characteristics of these two groups are shown in Table 6, with both groups showing statistical differences in albumin levels (lower albumin levels in the cavity group), ethnicity (African participants having a higher level of cavitation and Asians with a greater number without cavitation), TTP (lower TTP in the cavity group), and percentage of lung field affected (greater in the cavity group). HIV status, diabetes status, culture TTP (log_10_TTP), serum albumin, number of grade 3 or 4 TB symptoms, BMI, age, and ethnicity were found to have a statistically significant effect on the radiological severity of chest images by univariable analysis in the group with cavity disease (Table 7). In the non-cavitatory disease group, only ethnicity, serum albumin, and number of grade 3 symptoms were statistically significant on univariable analysis. Putting these significant variables in a multivariable regression model (Table 8) in those patients with cavitatory disease, the factors found to have a significant effect on the area of lung field affected were BMI, serum albumin, and log_10_TTP. In those without cavitatory disease, the factors found to have a significant effect on area of lung field affected were the number of grade 3 and 4 symptoms and serum albumin.

**Table 6:** Characteristics of those with and without cavitation used in the analysis comparing other baseline factors and radiological severity on CXR at diagnosis

	WITH CAVITY (*n* = 1049)			WITHOUT CAVITY (*n* = 305)			*p* value
Baseline characteristic	n	Median	IQR (1st, 3rd quartile)	n	Median	IQR	
Age (years)		31.4	23.7, 41.0		32.1	25.2, 44.2	0.03
Sex							0.95
Male	742 (70.7%)				217 (71.1%)		
Female	307 (29.3%)				88 (28.9%)		
Ethnicity							
African	742 (70.7%)				182 (59.7%)		< 0.01
Asian	307 (29.3%)				123 (40.3%)		
Diabetes							
Yes	50 (4.5%)				19 (6.2%)		0.38
No	1049 (95.5%)				286 (93.8%)		
HIV							0.79
Yes	81 (7%)				25 (8.2%)		
No	1081 (93%)				280 (91.8%)		
No. of Grade 3 + symptoms	68				16		0.51
Albumin (g/L)		33.5	9.0,37.8		37	32.0, 40.0	< 0.01
Body mass index (kg/m^2^)		18.3	16.9,20.1		19.0	17.3, 21.1	< 0.01
Time to positivity (hours)		112	86.0, 144.0		138	100, 188	< 0.01
Average area affected		20	13.75, 30		10	6.25, 16.9	< 0.01

**Table 7:** Results of univariable analysis. The β-coefficient represents the change in percentage area of lung field affected for every 1 unit increase in variable. For Log_10_TTP, this is the change in percentage area affected for every 10-fold increase in TTP

	With cavity			Without cavity		
	β-coefficient	95% CI	p value	B-coefficient	95% CI	p value
Age	0.09	(0.03, 0.15)	0.003	−0.0004	(−0.07, 0.07)	0.90
Sex						
Male vs. female	0.91	(−0.71, 2.5)	0.27	−0.41	(−2.49, 1.68)	0.70
Ethnicity						
Asia vs. Africa	−2.33	(−3.95, − 0.73)	0.004	− 2.5	(−4.41, − 0.59)	0.01
Diabetes	1.17	(−2.27, 4.63)	0.05	1.86	(−2.04, 5.77)	0.39
Positive vs. negative						
HIV						0.14
Positive vs. negative	2.11	(−0.67, 4.89)	0.01	−2.78	(−6.21, 0.65)	0.11
Albumin (g/L)	−0.72	(− 0.82, − 0.61)	< 0.001	−0.53	(− 0.68, − 0.38)	< 0.001
Body mass index	− 0.55	(− 0.76, − 0.33)	< 0.001	−0.13	(− 0.41, 0.19)	0.40
MGIT Log_10_ttp (hours)	−8.87	(−13.08, − 4.67)	< 0.001	− 2.41	(−7.10, 2.29)	0.31
Grade 3+ tuberculosis symptoms	2.17	(0.33, 4.02)	0.02	5.03	(1.88, 8.17)	0.002

**Table 8:** Multivariable regression analysis using variables found significant in univariate analysis. The β-coefficient represents the change in percentage area affected for every 1 unit rise in variables for albumin, BMI, number of grade 3/4 symptoms. and age. For log_10_TTP, this represents the change in percentage area affected for a 10-fold increase in TTP. For ethnicity, HIV, and diabetes this indicates the percentage difference in area affected between the two groups (for example, compared to the African cohort, Asians had 0.67% less area affected on the CXR than the African cohort)

	With cavity			Without cavity		
	β-coefficient	95% CI	*p* value	β-coefficient	95% CI	*p* value
Log_10_TTP (hours)	−4.72	(−8.73, 0.72)	0.02			
Albumin (g/L)	−0.65	(−0.76, −0.53)	< 0.001	−0.48	(− 0.64, − 0.32)	< 0.001
Grade 3 + Symptoms	0.05	(−1.68, 1.79)	0.95	3.13	(0.11, 6.15)	0.04
Body mass index	−0.50	(−0.72, − 0.27)	< 0.001	−0.07	(− 0.33, 0.19)	0.63
Ethnicity						
Asia vs. Africa	−0.67	(−2.33, 0.98)	0.43	−0.90	(−2.80, 1.00)	0.35
Age	0.05	(−0.01, 0.11)	0.13			
HIV						
Positive vs. Negative	0.54	(−2.09, 3.17)	0.69			
Diabetes						
Positive vs. Negative	3.03	(−0.33, 6.40)	0.07			

## Discussion and conclusions

The REMoxTB study provided a unique opportunity to address important questions about the role of radiology in the diagnosis and evaluation of severity of TB infection in a large group of smear- and culture-positive patients with PTB that spanned two continents. The subjectivity of CXR interpretation has been a longstanding concern in clinical practice and there have been multiple attempts to develop methods to standardize image reading in order to reduce reader variability [12, 40–42]. This study shows that agreement between readers in cavity assessment was moderate (Kappa score 0.495), comparable to other studies that found a Kappa agreement variation on cavity presence from 0.24 to 0.7 [43–48]. The clustering of scores across the x-axis and ‘0’ line of full agreement on the Bland–Altman plot (Fig. 2) confirms that the assessment of area of lung field affected is reproducible.

A high proportion of patients in this study had radiological evidence of cavitation (78%), compared with 72% in a study of 800 Turkish patients [20], 53.1% in a study of 893 USA-based patients [48], and 51% in a recent multicenter trial of 1692 patients in African sites [49]. Previous reports have suggested that the presence and number of cavities is related to bacterial load [12, 19–22, 50], but most of these studies were small, with an average of 138 patients (a range of 61–244). Using this large sample of patients we were able to show that there is a statistically significant reduction in the TTP (our surrogate for bacterial load) in patients with cavities compared to those without, with a median reduction of 26 h (*p* < 0.001). The large number of patients in this study provides the statistical power to demonstrate this unequivocally. It would, however, be reasonable to assume that such a reduction in TTP is of modest clinical significance, given that the replication rate of *M. tuberculosis* is approximately 14–24 h.

Looking at the two groups of cavity and no cavity, the cavity group had lower albumin, TTP, and greater area affected suggesting that those with cavities appear to have other markers of ‘severe’ disease.

More cavities were proportionately found in the African cohort than in the Asian cohort. This raises the question of whether ethnicity plays a role in cavity formation and the immune response to TB addressed in previous studies [29–31]. A recent study suggests that the pattern of radiological presentation at diagnosis is associated with certain inflammatory profiles in patients [30]. Significant differences between the cytokine response of Africans and Eurasian patients rather than *Mycobacterial* strain type have been demonstrated [30, 31]. This may be a contributing factor to the radiological severity of patients at presentation as we also noted a small but significant difference through univariate analysis of radiological score between patients of African origin and those of south and southeast Asian origin that was lost when put into a multivariable analysis.

The study also shows a relationship between overall area of lung field affected on radiograph and bacterial load with a very shallow association seen on the scatterplot presented (Fig. 4). The association described that it would require a 10-fold increase in TTP to change the area affected by 11%, suggesting that patients with a higher bacterial load do have greater radiological severity but the effect of this association is small.

Our study addresses the effect of variables such as ethnicity, initial bacterial load, nutritional status, HIV status, sex, age, symptom severity, and diabetic status by multivariable-regression analysis on radiological severity. When weighted against each other in a model, bacterial load does not have a statistically significant effect on the degree of diseased lung field on CXR in the group with non-cavitatory disease and, again, a modest effect in those with cavitation. This fits with the autopsy findings that Canetti described, where higher bacillary burden was found within cavities and their surrounding tissues but was much lower within the inflammatory, non-cavitating tissue alone [25].

**Fig. 5:**
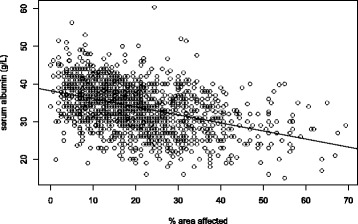
Scatterplot of the serum albumin levels (x-axis) and the percentage of lung field affected on the CXR (y-axis) for all 1354 participants. A linear regression line shows a steady decrease in serum albumin as more area is affected by disease

The only variable found to be related to the severity of the CXR at diagnosis is the serum albumin level in both the cavity and non-cavity groups. Poor nutritional status of patients with TB, using pre-treatment albumin levels and BMI as surrogate markers of nutritional status [33, 34, 51], has been associated with poorer treatment outcomes and death. In our study, patients with low serum albumin concentration at diagnosis (at a level of 15 g/L at the lowest) had a contributing effect to the radiological severity, but again to a modest degree, with a 0.65% and 0.48% decrease in area affected for every 1 g/L increase in serum albumin at baseline in the cavitatory and non-cavitatory groups, respectively (Fig. 5).

Through this analysis, our findings show that the factors affecting the appearance of the radiograph are likely to be multifactorial and to include host parameters such as ethnicity, age, co-morbidities, the bacterial load, and degree of disease progression. The interaction of the factors affecting the inflammatory response of an individual to PTB infection is being explored in other research.

We included those with HIV and type II diabetes mellitus in our cohort and found no significant effect on the radiological severity. This may be due to our HIV cohort being a select group with CD4 counts > 250 at PTB diagnosis without preceding anti-retrovirals and those with diabetes with less severe disease as a requirement for the clinical trial. They may, therefore, not reflect the full spectrum of morbidity and its effects on CXR severity.

In summary, our study is the largest review of radiology in a well characterized patient group with smear- and culture-positive PTB and suggests that, although CXR is a valuable tool for diagnosis, its use for judging the bacterial burden of disease has limited value. This is not unexpected, as the radiological image appears to be a composite of the interaction of disease pathology caused by the organism, the severity of the immune response and the nutritional status of the patient. The statistical power of this large study has enabled us to precisely measure the associations between CXR severity and other factors measured. The effect of serum albumin level on the radiological severity serves as an indicator that hypo-albuminemia is a marker of disease severity, as shown in other studies where it has been indicated to predict poor outcome in PTB [52]. The full value of CXR as a prognostic marker is yet to be seen and warrants further analysis. Although the associations between CXR severity and other factors conform with our expectations that patients with higher bacterial burden have more extensive disease, the small size of the effect and the finding that, in a multivariable model, it is outweighed by other patient factors in those without cavitation and is modest in those with cavitation would suggest that clinicians should be cautious in over-interpreting cause of radiological disease extent at diagnosis.

## Additional file

Additional file 1: List of ethics committee approving the REMoxTB study. (DOCX 894 kb)

## Abbreviations

AFB: acid fast bacilli
BMI: body mass index
CXR: chest x-ray
DICOM: digital imaging and communications in medicine
MGIT: mycobacteria-growth-indicator-tube
PTB: pulmonary tuberculosis
TB: tuberculosis
TTP: time-to-positivity

## References

[1] Garland LH (1948). Conditions to be differentiated in the roentgen diagnosis of pulmonary tuberculosis. Ann Intern Med.

[2] Cardinale L, Parlatano D, Boccuzzi F, Onoscuri M, Volpicelli G, Veltri A (2015). The imaging spectrum of pulmonary tuberculosis. Acta Radiol.

[3] Tattevin P (1999). The validity of medical history, classic symptoms, and chest radiographs in predicting pulmonary tuberculosis ^*^: derivation of a pulmonary tuberculosis prediction model. Chest J.

[4] Skoura E, Zumla A, Bomanji J (2015). Imaging in tuberculosis. Int J Infect Dis.

[5] World Health Organization (2013). Systematic Screening for Active Tuberculosis: Principles and Recommendations.

[6] World Health Organization. Chest radiography in tuberculosis detection: summary of current WHO recommendations and guidance on programmatic approaches. World Health Organization. 2016. http://www.who.int/iris/handle/10665/252424. ISBN 9789241511506.

[7] Burman WJ, Goldberg S, Johnson JL, Muzanye G, Engle M, Mosher AW (2006). Moxifloxacin versus ethambutol in the first 2 months of treatment for pulmonary tuberculosis. Am J Respir Crit Care Med.

[8] Benator D, Bhattacharya M, Bozeman L, Burman W, Cantazaro A, Chaisson R (2002). Rifapentine and isoniazid once a week versus rifampicin and isoniazid twice a week for treatment of drug-susceptible pulmonary tuberculosis in HIV-negative patients: a randomised clinical trial. Lancet.

[9] Simon G (1966). Radiology in epidemiological studies and some therapeutic trials. Br Med J.

[10] Fox AJ (1975). Classification of radiological appearance and the derivation of a numerical score. Br J Ind Med.

[11] National Tuberculosis and Respiratory Disease Association. Diagnostic Standards and Classification of Tuberculosis. New York: National Tuberculosis and Respiratory Disease Association; 1969. p. 94.

[12] Ralph AP, Ardian M, Wiguna A, Maguire GP, Becker NG, Drogumuller G (2010). A simple, valid, numerical score for grading chest x-ray severity in adult smear-positive pulmonary tuberculosis. Thorax.

[13] Olaru ID, Heyckendorf J, Grossmann S, Lange C (2014). Time to culture positivity and sputum smear microscopy during tuberculosis therapy. PLoS One.

[14] Padayatchi N, Gopal M, Naidoo R, Werner L, Naidoo K, Master I (2014). Clofazimine in the treatment of extensively drug-resistant tuberculosis with HIV coinfection in South Africa: a retrospective cohort study. J Antimicrob Chemother.

[15] Kenangalem E, Waramori G, Pontororing GJ, Sandjaja, Tjitra E, Maguire G (2013). Tuberculosis outcomes in Papua, Indonesia: the relationship with different body mass index characteristics between Papuan and non-Papuan ethnic groups. PLoS One.

[16] Pinto LM, Dheda K, Theron G, Allwood B, Calligaro G, van Zyl-Smit R (2013). Development of a simple reliable radiographic scoring system to aid the diagnosis of pulmonary tuberculosis. PLoS One.

[17] Rathman G, Sillah J, Hill PC, Murray JF, Adegbola R, Corrah T (2003). Clinical and radiological presentation of 340 adults with smear-positive tuberculosis in The Gambia. Int J Tuberc Lung Dis..

[18] Brust JCM, Berman AR, Zalta B, Haramati LB, Ning Y, Heo M (2013). Chest radiograph findings and time to culture conversion in patients with multidrug-resistant tuberculosis and HIV in Tugela Ferry, South Africa. PLoS One.

[19] Matsuoka S, Uchiyama K, Shima H, Suzuki K, Shimura A, Sasaki Y (2004). Relationship between CT findings of pulmonary tuberculosis and the number of acid-fast bacilli on sputum smears. Clin Imaging.

[20] Ozsahin SL, Arslan S, Epozturk K, Remziye E, Dogan OT (2011). Chest X-ray and bacteriology in the initial phase of treatment of 800 male patients with pulmonary tuberculosis. J Bras Pneumol.

[21] Perrin FMR, Woodward N, Phillips PPJ, McHugh TD, Nunn AJ, Lipman MCI (2010). Radiological cavitation, sputum mycobacterial load and treatment response in pulmonary tuberculosis. Int J Tuberc Lung Dis..

[22] Palaci M, Dietze R, Hadad DJ, Ribeiro FKC, Peres RL, Vinhas SA (2007). Cavitary disease and quantitative sputum bacillary load in cases of pulmonary tuberculosis. J Clin Microbiol.

[23] O’Sullivan DM, Sander C, Shorten RJ, Gillespie SH, Hill AVS, McHugh TD (2007). Evaluation of liquid culture for quantitation of Mycobacterium tuberculosis in murine models. Vaccine.

[24] Pheiffer C, Carroll NM, Beyers N, Donald P, Duncan K, Uys P (2008). Time to detection of Mycobacterium tuberculosis in BACTEC systems as a viable alternative to colony counting. Int J Tuberc Lung Dis..

[25] Canetti G (1955). The Tubercle Bacillus in the Pulmonary Lesion of Man.

[26] Aderaye G, Bruchfeld J, Assefa G, Feleke D, Källenius G, Baat M (2004). The relationship between disease pattern and disease burden by chest radiography, M. tuberculosis load, and HIV status in patients with pulmonary tuberculosis in Addis Ababa. Infection.

[27] Dooley KE, Chaisson RE (2009). Tuberculosis and diabetes mellitus: convergence of two epidemics. Lancet Infect Dis.

[28] Pérez-Guzman C, Torres-Cruz A, Villarreal-Velarde H, Salazar-Lezama MA, Vargas MH (2001). Atypical radiological images of pulmonary tuberculosis in 192 diabetic patients: a comparative study. Int J Tuberc Lung Dis..

[29] Morris CD (1989). The radiography, haematology and biochemistry of pulmonary tuberculosis in the aged. Q J Med.

[30] Coussens AK, Wilkinson RJ, Nikolayevskyy V, Elkington PT, Hanifa Y, Islam K (2013). Ethnic variation in inflammatory profile in tuberculosis. PLoS Pathog.

[31] Pareek M, Evans J, Innes J, Smith G, Hingley-Wilson S, Lougheed KE (2013). Ethnicity and mycobacterial lineage as determinants of tuberculosis disease phenotype. Thorax.

[32] Thorson A, Long NH, Larsson LO (2007). Chest X-ray findings in relation to gender and symptoms: a study of patients with smear positive tuberculosis in Vietnam. Scand J Infect Dis.

[33] Matos ED, Moreira Lemos AC (2006). Association between serum albumin levels and in-hospital deaths due to tuberculosis. Int J Tuberc Lung Dis..

[34] Kim H-J, Lee C-H, Shin S, Lee JH, Kim YW, Chung HS (2010). The impact of nutritional deficit on mortality of in-patients with pulmonary tuberculosis. Int J Tuberc Lung Dis..

[35] Hales CM, Heilig CM, Chaisson R, Leung CC, Chang KC, Goldberg SV (2013). The association between symptoms and microbiologically defined response to tuberculosis treatment. Ann Am Thorac Soc.

[36] Gillespie SH, Crook AM, McHugh TD, Mendel CM, Meredith SK, Murray SR (2014). Four-month moxifloxacin-based regimens for drug-sensitive tuberculosis. N Engl J Med.

[37] World Health Organization. Antiretroviral Therapy for HIV Infection in Infants and Children: Towards Universal Access. Recommendations for a Public Health Approach. 2010. ISBN 9789241599801. 23741772

[38] R Development Core Team. R: A Language and Environment for Statistical Computing. Vienna: R Foundation for Statistical Computing; 2014. https://www.r-project.org/.

[39] Mandrekar JN (2011). Measures of interrater agreement. J Thorac Oncol.

[40] Stout JE, Kosinski AS, Hamilton CD, Goodman PC, Mosher A, Menzies D (2010). Effect of improving the quality of radiographic interpretation on the ability to predict pulmonary tuberculosis relapse. Acad Radiol.

[41] Bossuyt PM (2003). The STARD Statement for Reporting Studies of Diagnostic Accuracy: explanation and elaboration. Clin Chem.

[42] Whiting P, Rutjes AWS, Reitsma JB, Bossuyt PMM, Kleijnen J (2003). The development of QUADAS: a tool for the quality assessment of studies of diagnostic accuracy included in systematic reviews. BMC Med Res Methodol.

[43] Balabanova Y, Coker R, Fedorin I, Zakharova S, Plavinskij S, Krukov N (2005). Variability in interpretation of chest radiographs among Russian clinicians and implications for screening programmes: observational study. BMJ.

[44] Abubakar I, Story A, Lipman M, Bothamley G, van Hest R, Andrews N (2010). Diagnostic accuracy of digital chest radiography for pulmonary tuberculosis in a UK urban population. Eur Respir J.

[45] Dawson R, Masuka P, Edwards DJ, Bateman ED, Bekker L-G, Wood R (2010). Chest radiograph reading and recording system: evaluation for tuberculosis screening in patients with advanced HIV. Int J Tuberc Lung Dis..

[46] Tudor GR, Finlay D, Taub N (1997). An assessment of inter-observer agreement and accuracy when reporting plain radiographs. Clin Radiol.

[47] Zellweger JP, Heinzer R, Touray M, Vidondo B, Altpeter E (2006). Intra-observer and overall agreement in the radiological assessment of tuberculosis. Int J Tuberc Lung Dis..

[48] Hamilton CD, Stout JE, Goodman PC, Mosher A, Menzies R, Schluger NW (2008). The value of end-of-treatment chest radiograph in predicting pulmonary tuberculosis relapse. Int J Tuberc Lung Dis..

[49] Merle CS, Fielding K, Sow OB, Gninafon M, Lo MB, Mthiyane T (2014). A four-month gatifloxacin-containing regimen for treating tuberculosis. N Engl J Med.

[50] Ors F, Deniz O, Bozlar U, Gumus S, Tasar M, Tozkoparan E (2007). High-resolution CT findings in patients with pulmonary tuberculosis: correlation with the degree of smear positivity. J Thorac Imaging.

[51] Khan A, Sterling TR, Reves R, Vernon A, Horsburgh CR, the Tuberculosis Trials Consortium (2006). Lack of weight gain and relapse risk in a large tuberculosis treatment trial. Am J Respir Crit Care Med.

[52] Kim S, Kim H, Kim WJ, Lee S-J, Hong Y, Lee H-Y (2016). Mortality and predictors in pulmonary tuberculosis with respiratory failure requiring mechanical ventilation. Int J Tuberc Lung Dis.

