# Supplementary figures and images for: Pretreatment chest x-ray severity and its relation to bacterial burden in smear positive pulmonary tuberculosis
Source: BMC Med. 2018 May 21;16:73. doi: 10.1186/s12916-018-1053-3 (PMC5961483; doi:10.1186/s12916-018-1053-3)

Additional file 1 List of ethics committee approving the REMoxTB study


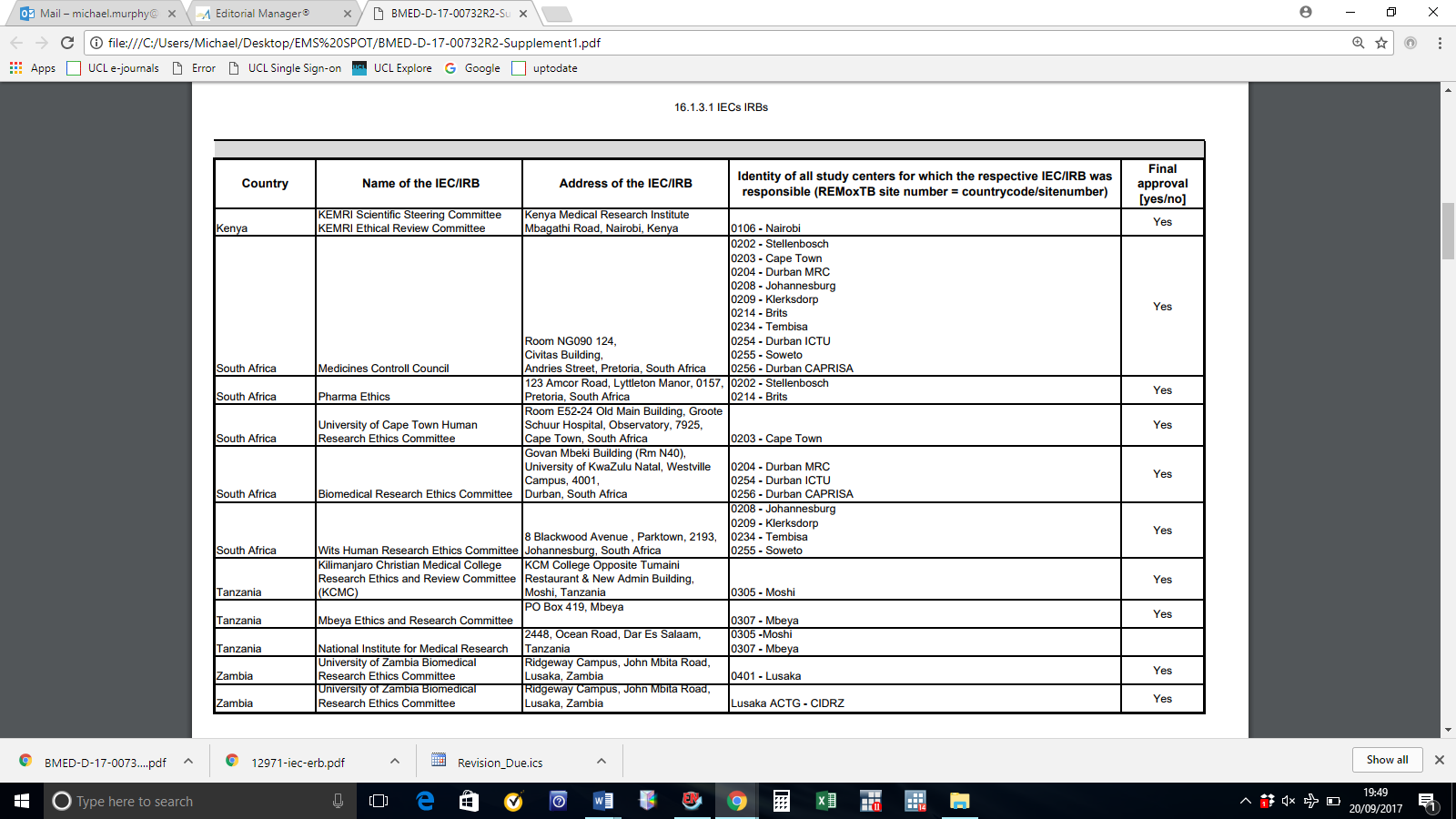


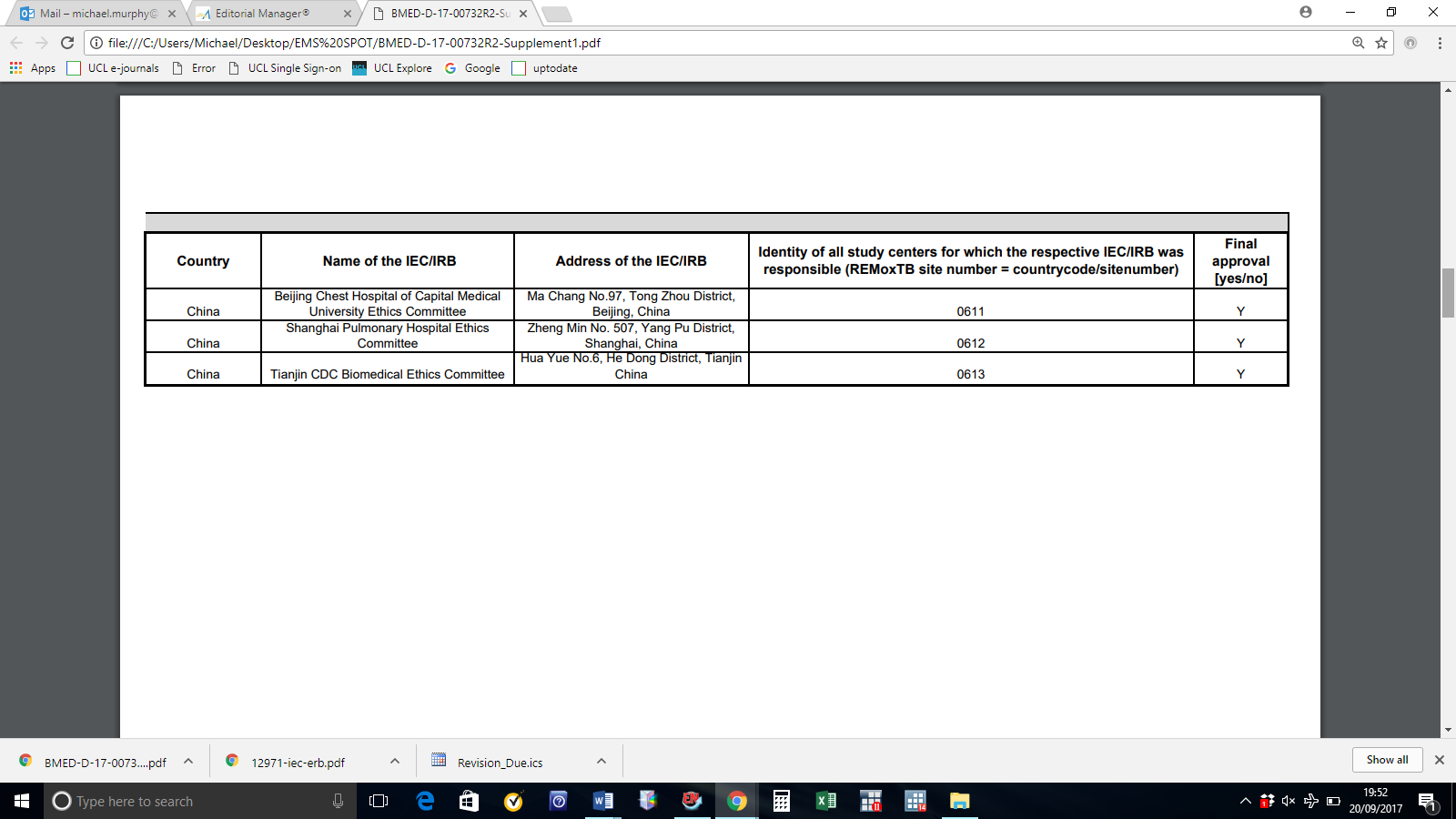


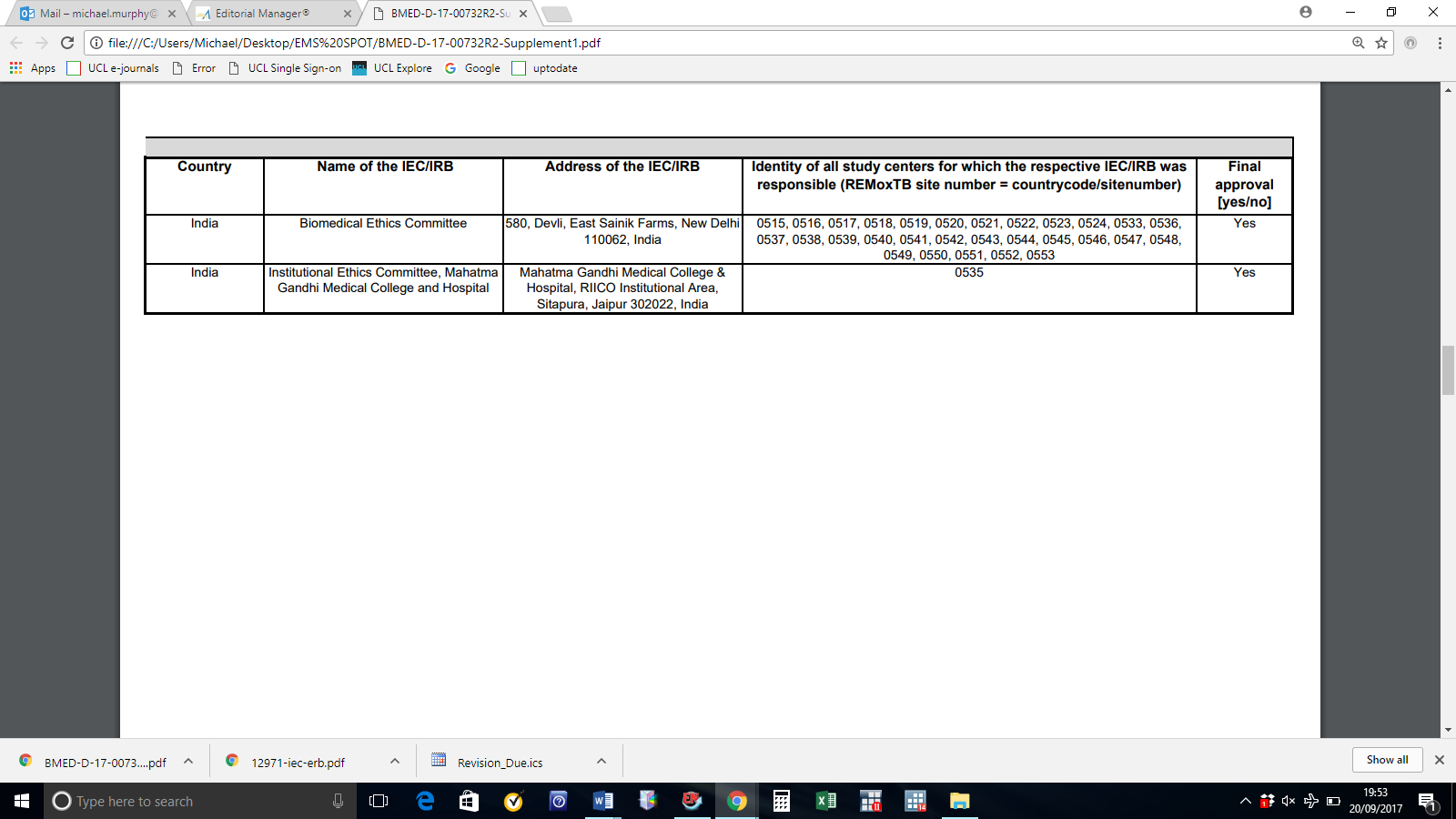


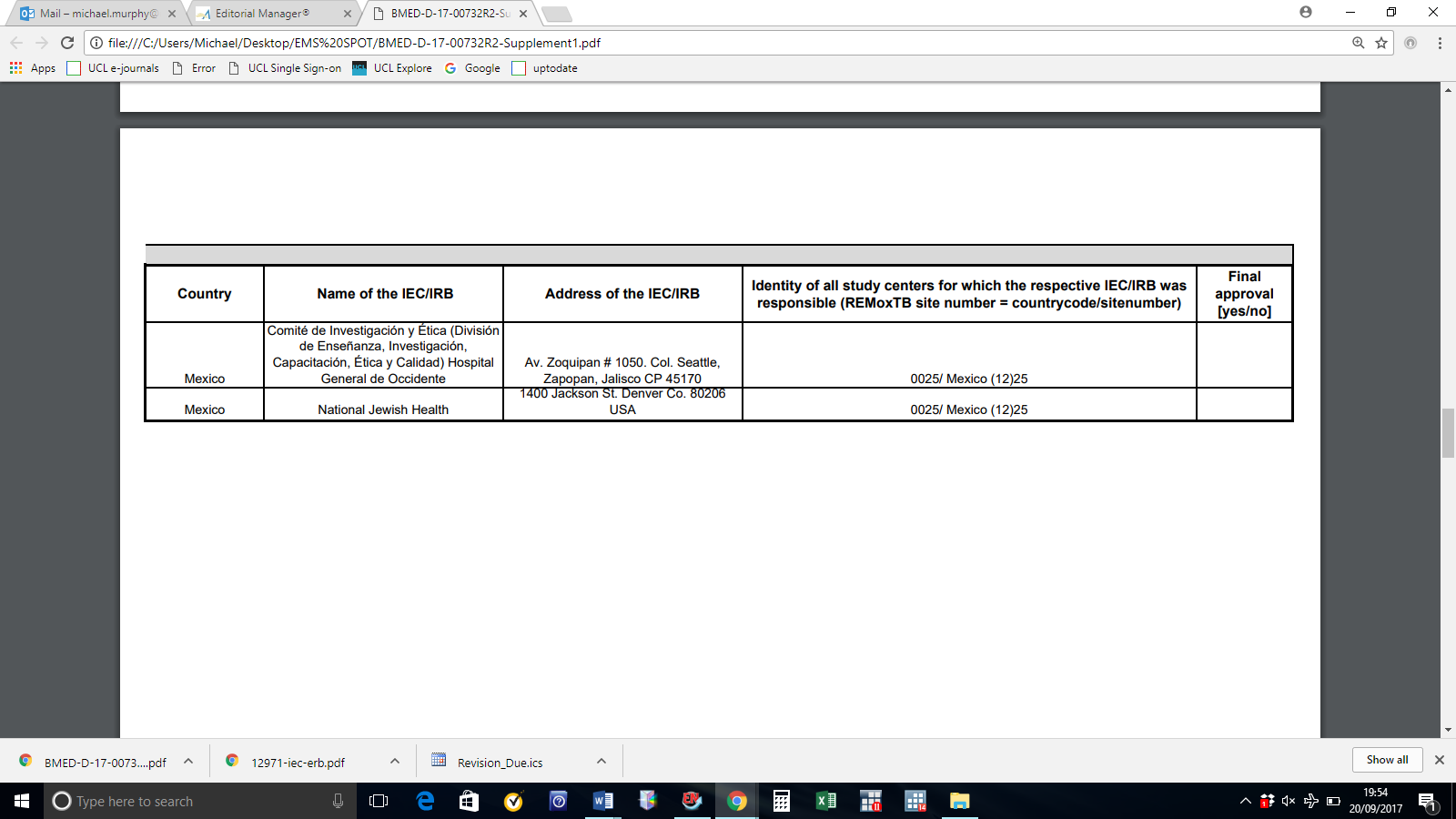


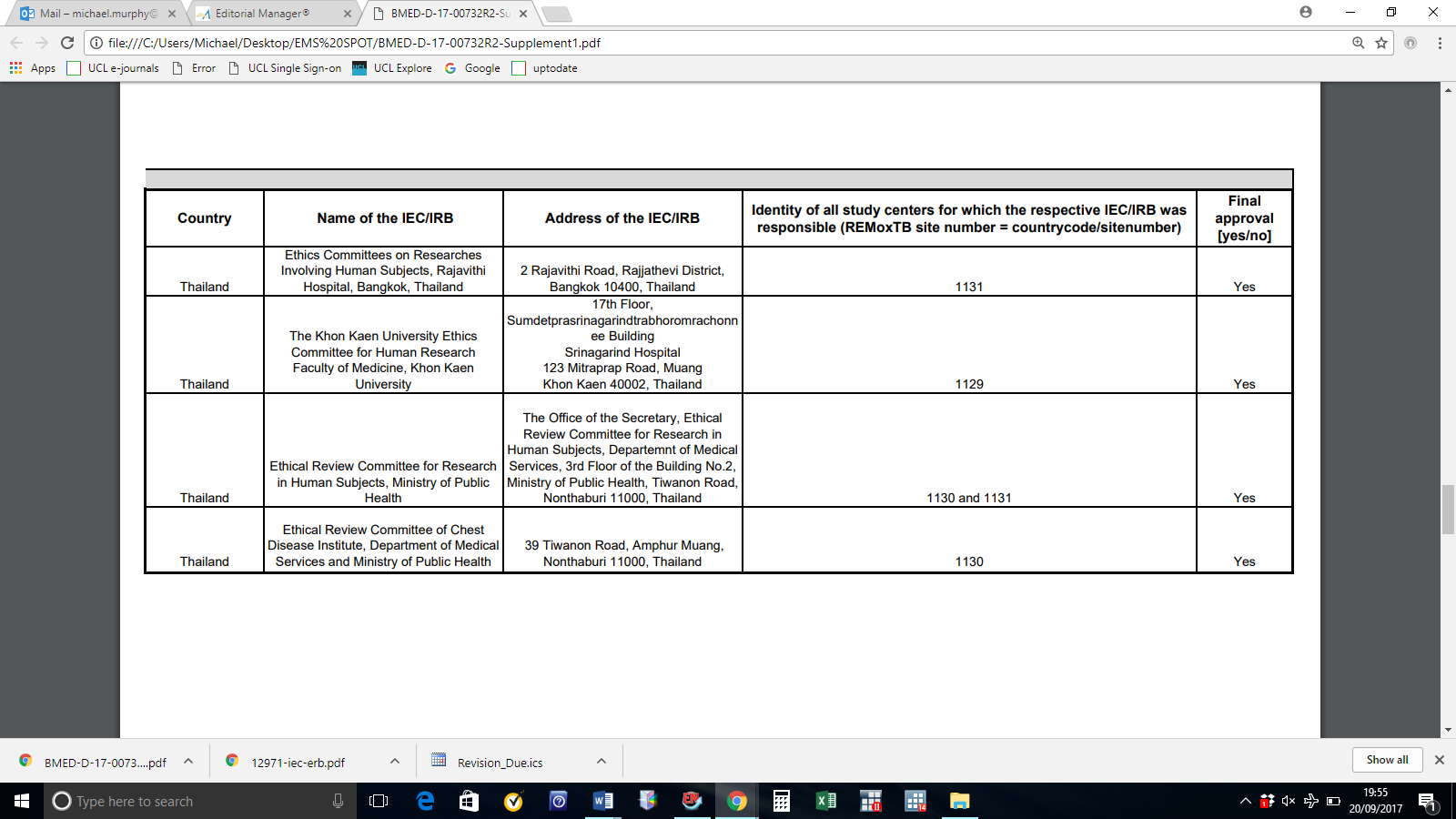


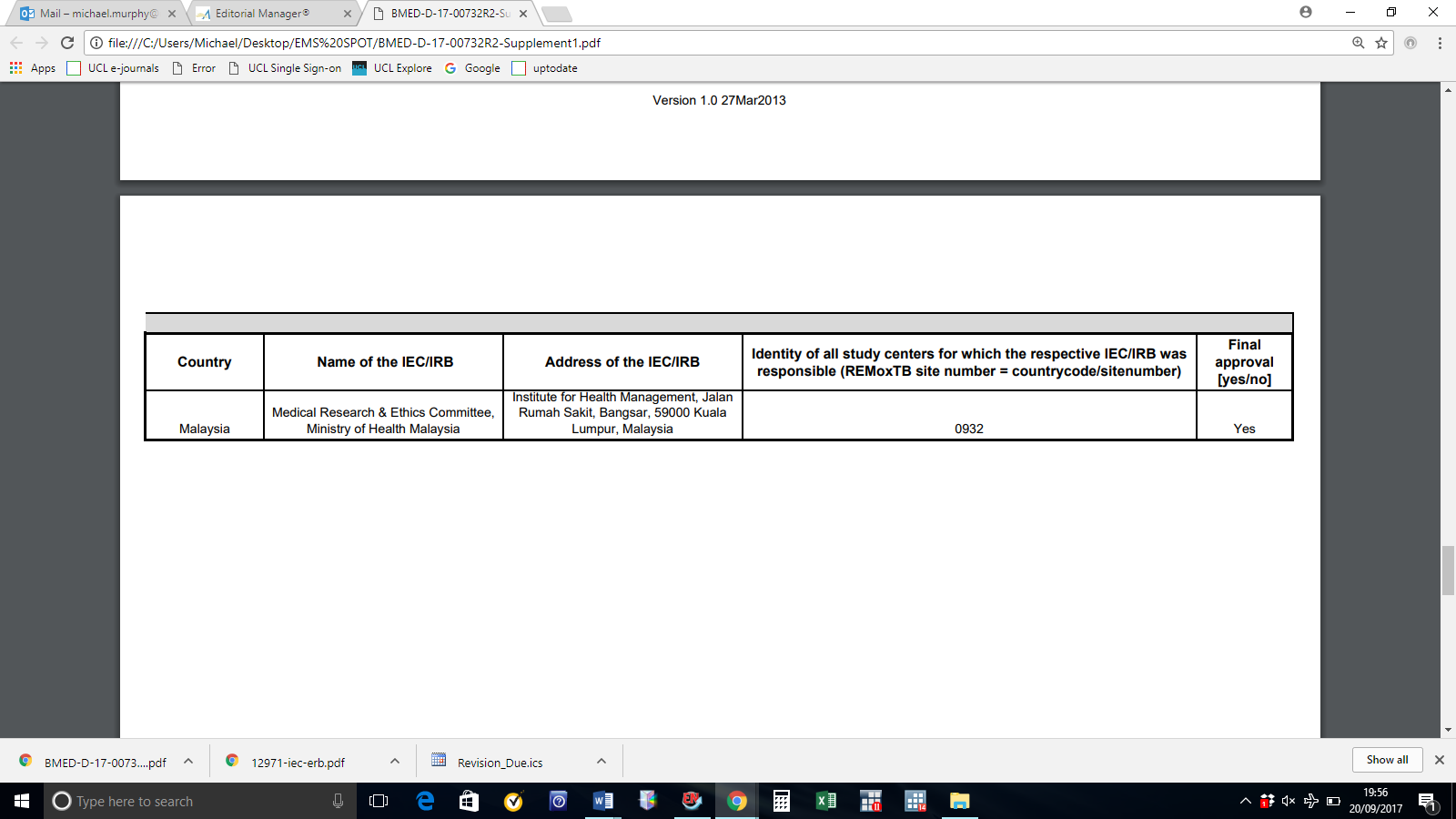

Supplement: Supplementary file 1 — List of ethics committee approving the REMoxTB study. (DOCX 894 kb) [file 12916_2018_1053_MOESM1_ESM.docx]
